# The Role of Lung Ultrasound Monitoring in Early Detection of Ventilator-Associated Pneumonia in COVID-19 Patients: A Retrospective Observational Study

**DOI:** 10.3390/jcm11113001

**Published:** 2022-05-26

**Authors:** Silvia Mongodi, Nello De Vita, Giulia Salve, Silvia Bonaiti, Francesco Daverio, Margherita Cavagnino, Gilda Siano, Alessandro Amatu, Giuseppe Maggio, Valeria Musella, Catherine Klersy, Rosanna Vaschetto, Belaid Bouhemad, Francesco Mojoli

**Affiliations:** 1 Anesthesia and Intensive Care, Fondazione IRCCS Policlinico San Matteo, DEA piano-1, Viale Camillo Golgi, 19, 27100 Pavia, Italy; nello.devita@uniupo.it (N.D.V.); silvi.bonaiti@gmail.com (S.B.); a.amatu@smatteo.pv.it (A.A.); g.maggio@smatteo.pv.it (G.M.); f.mojoli@smatteo.pv.it (F.M.); 2 Department of Translational Medicine, Università del Piemonte Orientale, Via Solaroli, 17, 28100 Novara, Italy; rosanna.vaschetto@med.uniupo.it; 3 Unit of Anaesthesia and Intensive Care, Department of Clinical-Surgical, Diagnostic, and Paediatric Sciences, Università di Pavia, Viale Brambilla, 74, 27100 Pavia, Italy; giulia.salve01@universitadipavia.it (G.S.); francesco.daverio01@universitadipavia.it (F.D.); margherita.cavagnino01@universitadipavia.it (M.C.); gildamaria.siano01@universitadipavia.it (G.S.); 4 Clinical Epidemiology and Biometrics, Fondazione IRCCS Policlinico S. Matteo, Viale Camillo Golgi, 19, 27100 Pavia, Italy; v.musella@smatteo.pv.it (V.M.); c.klersy@smatteo.pv.it (C.K.); 5 Department of Anaesthesiology and Intensive Care, C.H.U. Dijon, BP 77908, CEDEX, 21709 Dijon, France; belaid.bouhemad@psl.aphp.fr; 6 LNC UMR866, Department of Anesthesia and Surgical Critical Care, Université Bourgogne Franche-Comté, CEDEX, 21709 Dijon, France

**Keywords:** lung ultrasound, lung monitoring, ventilator-associated pneumonia, nosocomial infection, arborescent air bronchogram, lung ultrasound score, aeration quantification, aeration monitoring

## Abstract

Specific lung ultrasound signs combined with clinical parameters allow for early diagnosis of ventilator-associated pneumonia in the general ICU population. This retrospective cohort study aimed to determine the accuracy of lung ultrasound monitoring for ventilator-associated pneumonia diagnosis in COVID-19 patients. Clinical (i.e., clinical pulmonary infection score) and ultrasound (i.e., presence of consolidation and a dynamic linear–arborescent air bronchogram, lung ultrasound score, ventilator-associated lung ultrasound score) data were collected on the day of the microbiological sample (pneumonia-day) and 48 h before (baseline) on 55 bronchoalveolar lavages of 33 mechanically-ventilated COVID-19 patients who were monitored daily with lung ultrasounds. A total of 26 samples in 23 patients were positive for ventilator-associated pneumonia (pneumonia cases). The onset of a dynamic linear–arborescent air bronchogram was 100% specific for ventilator-associated pneumonia. The ventilator-associated lung ultrasound score was higher in pneumonia-cases (2.5 (IQR 1.0 to 4.0) vs. 1.0 (IQR 1.0 to 1.0); *p* < 0.001); the lung ultrasound score increased from baseline in pneumonia-cases only (3.5 (IQR 2.0 to 6.0) vs. −1.0 (IQR −2.0 to 1.0); *p* = 0.0001). The area under the curve for clinical parameters, ventilator-associated pneumonia lung ultrasound score, and lung ultrasound score variations were 0.472, 0.716, and 0.800, respectively. A newly appeared dynamic linear–arborescent air bronchogram is highly specific for ventilator-associated pneumonia in COVID-19 patients. A high ventilator-associated pneumonia lung ultrasound score (or an increase in the lung ultrasound score) orients to ventilator-associated pneumonia.

## 1. Introduction

Ventilator-associated pneumonia (VAP) is defined as hospital-acquired pneumonia in patients having received mechanical ventilation for >48 h; its incidence ranges from 5 to more than 20 cases per 1000 hospital admissions [1]. VAP is associated with a higher mortality rate and increased duration of mechanical ventilation and intensive care unit (ICU) length of stay [2].

A severe acute respiratory syndrome coronavirus 2 (SARS-CoV-2) infection, responsible for the coronavirus disease 2019 (COVID-19), may cause acute respiratory distress syndrome (ARDS). In the pandemic context, a high number of patients have been admitted to the ICU to start mechanical ventilation; thus, an increased number of patients have been at risk of developing VAP. Recent studies have reported a higher incidence of VAP in COVID-19 associated ARDS compared to ARDS with other etiologies [3,4], with a significant impact on mortality [5].

Lung ultrasound (LUS) [6], in particular with a quantitative approach and computation of the LUS score [7,8], can accurately assess lung aeration and can be daily performed to diagnose and monitor respiratory issues in critically ill patients [9,10,11,12]. In COVID-19 patients, it reliably stratifies the severity of lung involvement and allows monitoring the progression of the disease [13,14]. In this specific highly contagious disease, LUS reliably assesses COVID-19 disease when compared to a CT scan [15,16,17] and, therefore, has gained a leading position. As it is performed bedside, it also allows reducing the use of traditional radiology, minimizing the exposure of healthcare professionals to the virus [18]. LUS is therefore recommended by scientific societies and expert opinions as a first-line imaging tool for lung monitoring in COVID-19 patients [19,20,21,22,23].

The additional value of LUS in the diagnosis [24,25,26,27] and monitoring [28,29] of VAP has already been proposed; specific ultrasound signs combined with clinical parameters improve bedside early diagnosis and potentially reduce the introduction time to adequate antibiotic therapy [24,30,31]. In COVID-19 patients, the interest in an accurate bedside diagnostic tool is even higher than in standard ICU patients. LUS scores in COVID-19 (to monitor the progression of the disease [14,17,32,33] and detect a change in lung aeration associated with VAP [34]) have been described.

The present study aimed to test the accuracy of LUS signs and the modifications for the early diagnosis of VAP in COVID-19.

## 2. Material and Methods

A retrospective monocenter study of COVID-19 patients admitted to the ICU of a university hospital for acute respiratory failure, requiring mechanical ventilation, from 22 February to 22 December 2020. Ethical approval for this study was provided by the Ethics Committee (Comitato Etico Pavia, Pavia, Italy, ethics committee no. P-20210090349, chairperson: A. Gavazzi) in September 2021. Written consent for data collection was obtained according to national regulations.

### 2.1. Inclusion and Exclusion Criteria

Inclusion criteria were: 

Confirmed SARS-CoV-2-positive nasal swab and/or tracheal aspirate confirming the diagnosis of COVID-19 at admission to the ICU.

Age ≥ 18-year-old.

Invasive mechanical ventilation >48 h at the moment of microbiological sampling.

Two complete ultrasound examinations archived in the dedicated ultrasound picture archiving and communication system (uPACS): one on the day of the microbiological sample (VAP day) and a baseline examination performed 48 h before.

Exclusion criteria were:

Microbiological samples performed closer than 10 days after the first positive one (before a possible VAP resolution);

LUS incomplete examination (e.g., one of the two exams missing, incomplete storing of the clip (<12 per examination, pneumothorax preventing lung visualization in one or more regions).

### 2.2. Clinical Data

We collected the following data: age, gender, body mass index (BMI), procalcitonin (PCT), and C-reactive protein (CRP) blood levels. To calculate the clinical pulmonary infection score (CPIS) we collected: body temperature, white blood cell (WBC) count, presence of purulent tracheal secretions, the ratio of arterial oxygen partial pressure, and inspiratory oxygen fraction (PaO_2_/FiO_2_). Chest X-rays (CXR) were not performed routinely so we computed a simplified CPIS without CXR. All data were collected 48 h before (baseline) and on the same day of the microbiological sample (day of VAP diagnosis—VAP day). In addition, we collected the length of the mechanical ventilation, the ventilator-free day on day 28, and the ICU length of stay as outcome variables.

### 2.3. Lung Ultrasound

A complete 12-region examination was performed to monitor COVID-19 patients with a Vivid-iq ultrasound machine (GE Healthcare, Chicago, IL, USA). A 9-MHz linear probe was used if the pleural line was visualized, with focus on the pleural line, artifact-erasing software and harmonics abolished, and depth adjusted to at least twice the depth of the pleural line [8]; a phased array probe was used in case of consolidation or pleural effusion. Operators were either recognized experts in the field or trainees who completed 25 supervised examinations [35]. All clips were re-examined offline by a senior expert (SM, FM).

Two scores were computed, according to previous literature.

The LUS score was computed as follows [12,36,37]: a regional score was assigned to each of the six regions per hemithorax; score 0 for normal lung (A-lines or a maximum of two well-spaced B-lines), score 1 for moderate loss of aeration (≥3 well-spaced B-lines and/or artifacts occupying ≤50% of the visualized pleural line), score 2 for severe loss of aeration (artifacts occupying >50% of the pleural line), score 3 for complete loss of aeration (consolidation, predominant tissue-like pattern). The global LUS score was equal to the sum of the regional scores.

**Figure 1:**
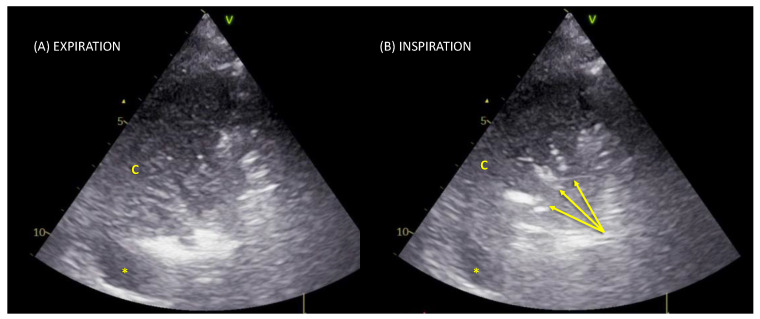
Lung ultrasound scan of a typical linear–arborescent air bronchogram. Posterior region in the transversal scan with a phased-array probe. Panel (**A**) in expiration, a tissue-like pattern corresponding to a consolidation (c) is visualized; a small pleural effusion is also visible as an anechoic space (*). Panel (**B**) during inspiration, a linear–arborescent air bronchogram appears (yellow arrows): a sign highly specific for ventilator-associated pneumonia.

The ventilator-associated pneumonia lung ultrasound score (VPLUS) was computed as follows: 1 point if there were ≥2 lung regions with subpleural consolidations, 2 points if there was ≥1 region with consolidation and a dynamic arborescent/linear air bronchogram (Figure 1; Video S1 SDC), and 1 point if there were purulent tracheal secretions [24].

LUS data were collected 48 h before (baseline) and on the same day as the microbiological samples (VAP day).

### 2.4. VAP Diagnosis

Distal microbiological samples were performed routinely once per week to monitor over-infections or in case of suspected VAP based on classical clinical parameters (fever/hypothermia, increased or purulent sputum, leukocytosis/leukopenia, and a further decline in oxygenation) [1]. They consisted of a bronchoalveolar lavage (BAL) performed with a fiberoptic bronchoscopy or in a blind mini-BAL performed with a sterile closed-loop aspiration system; this second sampling was considered acceptable in consideration of the high risks, e.g., contamination of healthcare providers, secondary to the airway openings in COVID-19 patients [23]. VAP was confirmed in case a pathogen was isolated in the specimen, with a cut-off value of 10^4^ colony forming units (CFU) or lower if antibiotics had been introduced within 48–72 h before the sampling [1].

### 2.5. Statistical Analysis

Quantitative and categorical variables are expressed as median (IQR) and number (percentage), respectively. Normal distribution was assessed by the Shapiro–Wilk test. According to the results of microbiological samples, the samples were divided into VAP cases and non-VAP cases. Patients were distinguished into VAP patients if they had developed at least one episode of VAP during the ICU stay, and non-VAP patients. Comparisons between the two populations were performed by the unpaired Wilcoxon–Mann–Whitney U-test for quantitative variables and Fisher’s exact test for categorical ones.

The receiver operator characteristic (ROC) curve analysis was performed to study the ability of ultrasound and clinical parameters to predict the diagnosis of VAP (areas under the curve (AUC) with the corresponding 95% confidence intervals). Cut-off points were obtained by the Youden index. Sensitivity, specificity, positive and negative predictive values, and positive and negative likelihood ratios with corresponding 95% confidence intervals were computed. With the analyzed 55 samples, it was possible to identify a difference in 0.8 standard deviations with a power of 80% and a type I error (two-way) of 5%. The *p*-value was considered significant if <0.05. The statistical analysis was performed by STATA14 for Macintosh.

## 3. Results

### 3.1. Population

Of the 306 COVID-19 patients admitted to our hospital’s ICU in the analyzed time frame, 47 were admitted to the unit where the uPACS was available for storing and reanalysis. These patients received 170 microbiological samples; of which, 41 were performed at admission and 74 were excluded because the ultrasound examinations were incomplete. A total of 55 samples of 33 patients were considered for final analysis according to the inclusion criteria; 26 were positive for VAP.

The 33 enrolled patients were mainly men 25 (75.8%), 62.0 (IQR 59.0 to 71.0) years old, and overweight (body mass index 28.7 (IQR 26.1 to 31.2) kg/m^2^) (Table 1). Overall mortality rate was 45.5%; median length of ICU stay was 30.0 (IQR 22.0 to 52.0) days. The median ventilator-free day on day 28 was 0.0 (IQR 0.0 to 0.0).

A total of 23 patients (69.7%) had at least one episode of VAP. No difference in mortality was observed between VAP and non-VAP patients (10 (43.5%) vs. 5 (50.0%); *p* = 0.730). However, VAP patients had longer ICU stays (38.0 (IQR 28.0 to 58.0) vs. 21.0 (IQR 17.0 to 30.0) days; *p* = 0.0135) and a longer need of mechanical ventilation (36.0 (IQR 25.0 to 56.0) vs. 21.0 (IQR 15.0 to 30.0) days; *p* = 0.0114). No difference was observed on the ventilator-free day on day 28.

Median length of mechanical ventilation before the microbiological sample was 13.0 (IQR 7.0 to 18.0) days; it was significantly longer in positive vs. negative samples (14.5 (IQR 11.0 to 23.0) vs. 10.0 (IQR 7.0 to 13.0); *p* = 0.0061). VAP pathogens isolated in the 26 positive samples were mainly Gram-negative bacteria, 23 (88.5%), followed by fungi, 3 (10.7%), and Gram-positive bacteria, 2 (7.7%), as displayed in Table 2; 2 were polymicrobial infections (E. Faecalis + A. Baumannii and P. Aeruginosa + A. Baumannii); 3 patients had 2 episodes of VAP. 

### 3.2. Baseline Findings

Clinical baseline findings were not different in VAP and non-VAP cases in terms of the need for oxygen supply, hypoxemia, WBC, and temperature. A higher prevalence of purulent secretions was also observed in VAP cases 48 h before the positive sample (6 (23.1%) vs. 0 (0.0%); *p* = 0.008). The CPIS score was not different in VAP and non-VAP cases.

Ultrasound findings were similar in VAP and non-VAP cases in terms of LUS score, VPLUS, number of regions presenting subpleural consolidations, and number of consolidations with dynamic linear–arborescent air bronchograms. A higher number of consolidated regions was observed in non-VAP cases (1.0 (IQR 0.0 to 2.0) vs. 0.0 (IQR 0.0 to 1.0); *p* = 0.0279).

### 3.3. VAP Day Findings

On the day the microbiological sample was collected, in VAP cases, patients were more hypoxemic (PaO_2_/FiO_2_ 164.0 (IQR 126.0 to 198.0) vs. 192.0 (IQR 165.0 to 220.0) mmHg; *p* = 0.0568), had higher WBC (13.7 IQR 11.0 to 15.8) vs. 11.5 (IQR 10.5 to 13.4) cells × 1000/mL; *p* = 0.0296), higher body temperatures (37.6 (IQR 37.0 to 38.1) vs. 37.0 (IQR 36.4 to 37.3) °C; *p* = 0.0155), and more frequently presented purulent secretions (18 (69.2%) vs. 7 (24.1%); *p* = 0.001). However, both the CPIS and the CPIS variations from baseline were similar in the two groups. The PCT variation was also not significantly different between VAP and non-VAP cases.

Ultrasound findings were significantly different in VAP and non-VAP cases: all patients with VAP presented at least 1 consolidated region (24/26), or >2 regions with subpleural consolidations. A higher median number of consolidated regions was observed in VAP (2.0 (IQR 1.0 to 3.0) vs. 0.0 (IQR 0.0 to 1.0); *p* = 0.0002), with a higher prevalence of new consolidations (2 (7.7%) vs. 1 (3.5%); *p* < 0.001). Within consolidations, newly appeared dynamic linear–arborescent air bronchograms were observed in VAP only (13 (50.0%) vs. 0 (0.0%); *p* < 0.0001). The VPLUS score was higher in VAP cases (2.5 (IQR 1.0 to 4.0) vs. 1.0 (IQR 1.0 to 1.0) points; *p* < 0.0001); accordingly, the VPLUS variation from baseline was higher in VAP cases; in particular, no increase was observed in non-VAP cases.

The LUS score was higher in VAP cases (18.5 (IQR 15.0 to 22.0) vs. 14.0 (10.0 to 18.0) points; *p* = 0.0173); when compared to the baseline value, in non-VAP cases, the change of the LUS score was in a median negative (−1.0 (IQR −2.0 to 1.0), corresponding to an improvement in lung aeration, while in VAP cases it increased in the median of 3.5 [2.0–6.0] points (*p* < 0.0001), corresponding to a deterioration of lung aeration.

### 3.4. Diagnostic Performances

Sensitivity, specificity, and AUC with corresponding 95% confidence intervals were computed for clinical and ultrasound findings.

The most relevant clinical parameter was the presence of purulent secretions with a sensitivity of 69.2%, specificity of 75.9%, and an AUC of 0.73 (95% CI 0.61 to 0.85).

The CPIS showed an AUC for VAP diagnosis of 0.588 (95% CI 0.437 to 0.738) with an optimal cut-off point identified at ≥5 (sensitivity 73.1%, specificity 55.2%). The CPIS variation AUC was 0.472 (95% CI 0.319 to 0.624) with an optimal cut-off point ≥0 (sensitivity 96.2%, specificity 10.3%).

The VPLUS AUC for VAP diagnosis was 0.798 (95% CI 0.693 to 0.902), significantly higher than the CPIS (*p* = 0.0084) and similar to purulent secretions (*p* = 0.1292), with an optimal cut-off point identified at ≥3 (sensitivity 50.0%, specificity 100.0%). The VPLUS variation AUC was 0.716 (95% CI 0.582 to 0.850), with an optimal cut-off point ≥2 (sensitivity 42.3%, specificity 100.0%).

**Figure 2:**
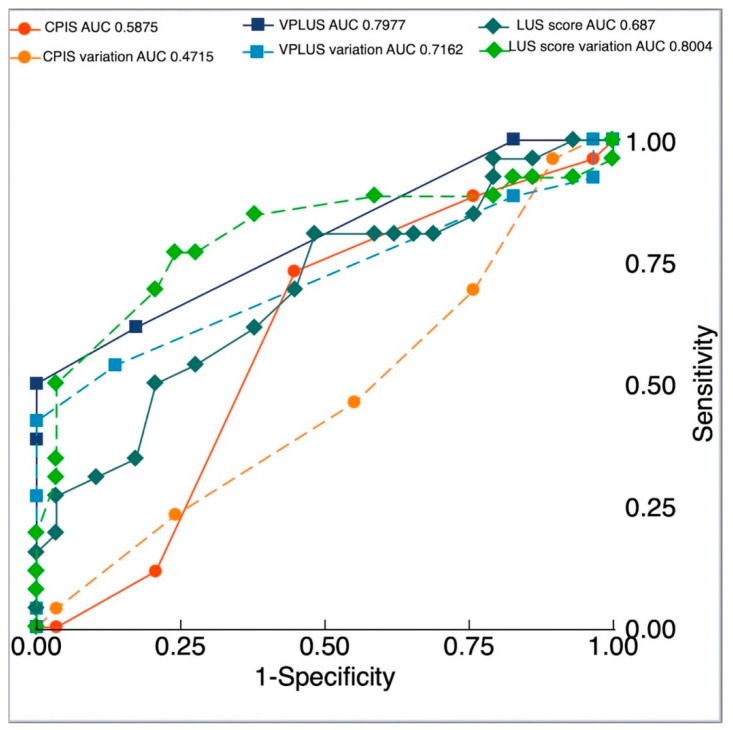
Diagnostic accuracy for clinical and ultrasound parameters, areas under the receiver operating characteristic curves generated for clinical pulmonary infection score (CPIS), CPIS variation, ventilator-associated pneumonia lung ultrasound score (VPLUS), VPLUS variation, lung ultrasound (LUS) score, and LUS score variation.

The LUS score AUC for VAP diagnosis was 0.687 (95% CI 0.545 to 0.829), with an optimal cut-off value ≥15 (sensitivity 80.8%, specificity 51.7%). The LUS score variation AUC was 0.8004 (95% CI 0.674 to 0.927), not different from VPLUS and purulent secretion diagnostic performances (*p* = 0.9693 and *p* = 0.3661, respectively). The optimal cut-off point was ≥2 (sensitivity 76.9%, specificity 75.9%). ROC curves are shown in Figure 2.

Combining the findings in a post hoc score, giving 1 point to the newly appeared linear/arborescent air bronchogram, 1 point if there was an increase in the LUS score ≥1, and 1 point if there were purulent secretions, AUC rose to 0.855 (95% CI 0.757 to 0.953), with an optimal cut-off point ≥2 (sensitivity 65.0%, specificity 93.0%).This combination of ultrasound and clinical parameters performs significantly better than purulent secretions alone (*p* = 0.0034).

## 4. Discussion

The main findings of the present work are: (1) The ventilator-associated pneumonia lung ultrasound score is highly accurate for the bedside early diagnosis of VAP in COVID-19. (2) The presence of a newly appeared dynamic linear–arborescent air bronchogram is 100% specific for the diagnosis of VAP. (3) An increase in the LUS score could orient to a VAP.

VAP is a common issue in critically ill patients and affects 5–40% of patients receiving invasive mechanical ventilation [38] with a very high incidence in trauma [39] and brain-injured patients [40]. VAP is associated with a prolonged duration of mechanical ventilation and ICU stay; however, attributable VAP mortality is still a matter of debate. To address this point, different methods have been used and the risk of death attributable to VAP has shown wide variability, according to the underlying disease [41] and pathogen identity [42,43]. In COVID-19, higher mortality associated with VAP was recently demonstrated [5].

Diagnosis of VAP is still a challenge and clinical suspicion of VAP is crucial for diagnosis and specific treatment initiation. According to European guidelines, distal quantitative samples should be obtained, and specific antimicrobial treatments should start when microbiological diagnoses are made. However, the time from sampling to pathogen identification usually ranges from 24 h for preliminary data to 48 h for precise identification; in this time-lapse, the physician has to decide whether to start empirical antimicrobial treatment or wait for microbiological confirmation. Overuse of broad-spectrum empirical treatment allows to start therapy earlier but can lead to antibiotics overuse and the development of multi-drug-resistant pathogens. By contrast, waiting for microbiological sample results may delay appropriate treatment and increase mortality.

In addition to clinical signs of infection, e.g., fever, leukocytosis, and decline in oxygenation, almost all definitions for VAP suspicion and diagnosing encompass imaging criteria. CXR is the most frequently used tool, but it is neither sensitive nor specific to VAP diagnosis, especially when a portable chest radiograph is used [44] and in patients already having pneumonia.

Lung imaging can be improved by using computed tomography; however, a systematic lung CT-scan approach poses multiple limitations, especially in a pandemic context characterized by increased risks and workloads for healthcare providers [18] and increased risks surrounding patient safety during transport. More recently, LUS was proposed as a reliable diagnostic tool for community-acquired pneumonia in emergency departments [45]. Although LUS has good sensitivity and specificity in detecting lung consolidations in ventilated patients, these conditions are very common in ICUs and are not specific to VAP [25,46]. Consolidations are almost constant in ICU patients in posterior regions and are potentially due to other causes, such as de-recruitment, atelectasis, and previous unsolved pulmonary conditions. However, according to Zhou and coworkers, LUS remains an interesting tool to assist in VAP diagnosis [27].

Interest in air bronchogram assessment was raised by Berlet et al. [26] and confirmed by Mongodi et al. [24]. In this latter study, two signs were associated with VAP: subpleural consolidations and dynamic linear/arborescent air bronchograms within lobar/hemilobar consolidations, the first being highly sensitive (81%) and the second highly specific (97%). A post hoc score for the diagnosis of VAP was tested, the ventilator-associated lung ultrasound score (VPLUS). The VPLUS showed an AUC of 0.743 with an optimal cut-off point at VPLUS ≥2 with specificity at 69% (50–84%) and sensitivity at 71% (58–81%), thus being significantly more accurate for VAP diagnosed when compared to standard clinical and radiological approaches. A recent work underlined the interest in air bronchogram assessments, and other post hoc scores, including LUS and pentraxin-3 levels, were tested. A LUPPIS score >7 was more accurate in VAP diagnosis compared to CPIS >6 (AUC 0.952, 84% sensitivity and 87.7% specificity vs. AUC 0.822, 44% sensitivity and 83% specificity) [47]. A similar score was also recently applied in neonates and the LUS accuracy was confirmed in this context (AUC 0.91, 94% sensitivity, and 67% specificity) [48]. Besides the diagnostic application, the changes in air bronchograms have been investigated as promising prognostic tools. A recent study suggested that the evolution of air bronchograms might be used to guide antibiotic escalation and to predict favorable clinical outcomes in children with community-acquired pneumonia [49].

In addition, the potential role of LUS as a monitoring tool (in addition to punctual assessment) was investigated [26]. LUS finding modifications, e.g., induced by disobstructive fiberoptic bronchoscopy, have been suggested to unmask the etiology of consolidations [31].

Finally, the role of LUS in VAP is not only limited to diagnoses but it may also be used to monitor the efficacy of antibiotics [29] and identify complications, such as empyema and abscesses [28].

In our population, we prospectively applied the LUS examination to patients with ARDS for lung monitoring. We then retrospectively collected data on the clinical and ultrasound features of VAP day when compared to baseline features 48 h before. Concerning clinical data, there were significant differences in many parameters in VAP cases on the day of the VAP (higher leukocytosis, temperature, purulent secretions); however, this was not translated into differences in the CPIS score, probably because these differences were statistically significant but too clinically subtle. In COVID-19 patients, all these clinical parameters can also be affected by viral pneumonia itself and by immunomodulating treatments, such as steroids or immunosuppressive drugs. Interestingly, the presence of purulent secretions reached a good diagnostic performance and a good negative predictive value; however as suggested by the international guidelines [1], VAP diagnosis implies lung parenchymal involvement. The presence of a pathogen in the upper airway without at least one imaging criteria is not considered enough for VAP diagnosis [1]; caution should be even higher when a qualitative parameter as purulent aspect of secretion is used for the diagnosis since it may be operator dependent. Our data show in fact that the combination of this simple parameter to ultrasound findings as an increase in LUS score and a newly appeared linear-arborescent air-bronchogram significantly improves the bedside diagnostic accuracy.

For what concerns ultrasound parameters, the VPLUS confirmed its higher accuracy in identifying VAP cases when compared to CPIS. The interest of linear /arborescent air-bronchogram as an extremely specific sign for VAP is confirmed. On the contrary, the presence of subpleural consolidations was here less useful in VAP diagnosis, since they are common signs in COVID-19 pneumonia or of its complications (i.e., peripheral infarction in pulmonary embolism [15,16,17]). In addition to what already known about LUS for early diagnosis of VAP, this study gives a clue on the interest in lung monitoring with the LUS score. An increase in both VPLUS and LUS score were in fact observed in VAP cases. The LUS score is not specific for VAP and allows monitoring overall lung aeration; in patients with clinical signs of pneumonia as purulent secretions, in case of a deterioration of lung aeration assessed with LUS score, a VAP should be suspected. The specificity of linear/arborescent air-bronchogram and the increase in LUS score or in VPLUS are more solid results than the simple increase in the number on consolidated regions, which is statistically significant but concerns a minority of patients. The presence of a consolidation is in fact know to be a non-specific sign for ventilator-associated pneumonia [27].

This study presents multiple limitations. First, being a retrospective observational monocentric study with a small sample size, it cannot be considered a definitive study, but it may guide prospective multicentric and adequately-powered works. The limited number of patients also justifies the fact that no difference could be demonstrated in important outcomes, such as mortality or ventilator-free days. Moreover, the mortality of the presented population is high—higher than the overall COVID-19 population admitted to our ICUs in the same timeframe; therefore, a selection bias of the most severe patients may be present.

Due to the high risk of contamination, a conventional BAL was not always performed, but a blind mini-BAL with a closed-loop aspiration system. However, this technique is considered acceptable since no difference in terms of accuracy has been suggested between BAL and endotracheal aspirate [50,51,52]; moreover, this makes the results closer to current clinical practices for COVID-19 management. It could be argued that the positivity of the distal microbiological sample alone is not enough for the diagnosis of pneumonia, which requires the demonstration of a parenchymal involvement [1]. However, demonstrating that the parenchymal involvement is due to an over-infection may be particularly challenging when the patient is admitted to the ICU for viral pneumonia; moreover, all VAP cases actually presented lung involvement at LUS, with at least one consolidated region or multiple regions with subpleural consolidations. We did not perform CXR systematically in acute respiratory failure patients [18], since it showed lower reliability than LUS. Moreover, during COVID-19, this allowed reducing exposure to healthcare workers; thus, CPIS was calculated without imaging findings. However, in the pandemic context, this approach seems closer to real clinical practices.

Fourth, this population did not always present all clinical features, suggesting that VAP and pre-test probability for CPIS were lower than in previous studies. Finally, due to the pandemic context, exams were performed by different operators at different levels of expertise [13], and no previous interobserver agreement study was performed. Nevertheless, the LUS interpretation, in particular, advanced skills such as identification of the dynamic linear/arborescent air bronchogram, was performed by expert operators (SM, FM; academic teachers involved in research in the field) through an offline analysis.

## 5. Conclusions

A lung ultrasound is confirmed to be a valuable tool in the bedside identification of patients with VAP, as well as in a difficult population already admitted to ICUs for pneumonia and ARDS. A newly appeared dynamic linear–arborescent air bronchogram is highly specific for VAP. A high VPLUS or an increase in the LUS score orients to VAP diagnosis.

## Figures and Tables

**Table 1:** Characteristics of the enrolled population of patients.

	Overall (33 Patients)	Non-VAP (10 Patients)	VAP (23 Patients)	*p*-Value
Males—*n* (%)	25 (75.8)	7 (70.0)	18 (78.3)	0.611
Age—years	62.0 [59.0–71.0]	73.0 [60.0–75.0]	61.0 [58.0–69.0]	0.0774
BMI—kg/m^2^	28.7 [26.1–31.2]	28.0 [27.3–29.4]	29.4 [26.1–31.8]	0.8754
SAPS II—points	46.0 [33.0–55.0]	48.0 [45.0–59.0]	45.0 [32.0–51.0]	0.2394
Length of stay in ICU—days	30.0 [22.0–52.0]	21.0 [17.0–30.0]	38.0 [28.0–58.0]	**0.0135**
Length of mechanical ventilation before the microbiological sample—days	13.0 [7.0–18.0]	10.0 [7.0–13.0]	14.5 [11.0–23.0]	**0.0061**
Overall length of mechanical ventilation—days	30.0 [22.0–50.0]	21.0 [15.0–30.0]	36.0 [25.0–56.0]	**0.0114**
Ventilator-free day on day 28—days	0.0 [0.0–0.0]	0.0 [0.0–0.0]	0.0 [0.0–0.0]	0.1357
Mortality in ICU—*n* (%)	15 (45.5)	5 (50.0)	10 (43.5)	0.730

Values are expressed as *n* (%) or median [interquartile range]—VAP: ventilator-associated pneumonia; BMI: body mass index; SAPS II: simplified acute physiology score; ICU: intensive care unit. Significant *p*-Values in bold.

**Table 2:** A total of 28 pathogens identified in 26 positive microbiological samples.

Identified Pathogens	*n* (%)
GRAM NEGATIVE BACTERIA	
Pseudomonas aeruginosa	9 (32.1)
Acinetobacter baumanii MDR	8 (28.6)
Klebsiella pneumoniae MDR	3 (10.7)
Enterobacter asburiae	1 (3.6)
Stenotrophomonas maltophilia MDR	1 (3.6)
Achromobacter xylosoxidans	1 (3.6)
GRAM POSITIVE BACTERIA	
Enterococcus faecalis	1 (3.6)
Corynebacterium striatum	1 (3.6)
FUNGI	
Aspergillus fumigatus	3 (10.7)

Values are expressed as *n* (%); MDR: multi-drug resistant.

## Data Availability

The dataset used during the current study is available from the corresponding author upon reasonable request.

## Supplementary Materials

The following supporting information can be downloaded at: https://www.mdpi.com/article/10.3390/jcm11113001/s1, Video S1: Lung ultrasound scan of a posterior region in a transversal scan with a phased-array probe. During inspiration, a linear–arborescent air bronchogram appears within a consolidated lobe. This pattern is highly specific for ventilator-associated pneumonia.
